# Calcium/Calmodulin-Dependent Serine Protein Kinase (*CASK*) Gene Polymorphisms in Pigeons

**DOI:** 10.3390/ani13132070

**Published:** 2023-06-22

**Authors:** Andrzej Dybus, Hanna Kulig, Wilhelm Grzesiak, Justyna Domke, Yu-Hsiang Yu, Yeong-Hsiang Cheng

**Affiliations:** 1 Department of Genetics, West Pomeranian University of Technology, Aleja Piastów 45, 70-311 Szczecin, Poland; adybus@zut.edu.pl (A.D.); justadomke@wp.pl (J.D.); 2 Laboratory of Biostatistics, Department of Ruminant Science, West Pomeranian University of Technology, Klemensa Janickiego 29, 71-270 Szczecin, Poland; wilhelm.grzesiak@zut.edu.pl; 3 Department of Biotechnology and Animal Science, National Ilan University, No.1, Sec. 1, Shennong Rd., Yilan City 26047, Taiwan; yuyh@niu.edu.tw (Y.-H.Y.); yhcheng@ems.niu.edu.tw (Y.-H.C.)

**Keywords:** *CASK* gene, homing pigeons, genetic markers, racing performance

## Abstract

**Simple Summary:**

The aim of the study was to detect the nucleotide sequence variability in the calcium/calmodulin-dependent serine kinase (*CASK*) gene in pigeons. One of the detected polymorphic sites (g.8893G > A) located at position −3 relative to the start codon was selected for genotyping in 517 pigeons and was used to establish possible associations between genotypes and flight performance of pigeons. Frequencies of the *AA* genotype were higher in homing pigeon groups than in the group of non-homing pigeons. The results can be treated as a contribution to further research that could confirm the functionality of the analysed SNP in shaping the sports phenotype of domestic pigeons.

**Abstract:**

Calcium/calmodulin-dependent serine protein kinase (CASK) is an multidomain protein involved in tissue development and cell signalling. In skeletal muscle, it is involved in the development of neuromuscular junctions. The participation of a pigeon in racing is a great physical effort that causes many changes in the skeletal muscles. Thus, the purpose of the study was to detect the nucleotide sequence variability in the calcium/calmodulin-dependent serine kinase (*CASK*) gene in domestic pigeons (*Columba livia domestica)* and assess the potential impact of DNA polymorphisms on the flight performance of pigeons. The research included a total of 517 individuals. DNA was extracted from the blood. A DNA fragment from nucleotides 8689 to 9049 of the *CASK* (NW_004973256.1 sequence) of six unrelated pigeons were sequenced. One of the detected polymorphic sites (g.8893G > A), located a very close to the start codon, was selected for genotyping in all individuals. The association studies included a total of 311 young homing pigeons that participated in racing competitions. The homing pigeons showed higher frequencies of the *AA* genotype than non-homing ones (*p* < 0.05). In rock pigeons only the *GG* genotype was found. Further research could confirm the functionality of the *CASK* g.8893G > A SNP in shaping the racing phenotype of pigeons, and the *AA* genotype could be useful as a selection criterion in pigeon breeding.

## 1. Introduction

Calcium/calmodulin-dependent serine protein kinase (CASK) is an evolutionarily conserved multidomain protein involved in scaffolding the synapse, organizing ion channels, and regulating neuronal gene transcription [1,2,3]. CASK is able to bind neurexin [4] and the interaction (CASK-neurexin) stabilizes structures at neuronal synapses [5]. The importance of CASK was manifested in KO mutant mice; they died shortly after birth [6]. The protein is required for mouse survival, but normal electrical properties and normal synapse formation was observed in neurons lacking CASK. Interestingly, CASK-deficient neurons were characterised by increased glutamatergic spontaneous synaptic release, and decreased GABAergic synaptic release [7]. 

In skeletal muscle, CASK is involved in the development of neuromuscular junctions (NMJs) [8,9,10]. NMJs are the sites of the transmission of electrical stimuli from nerve to the muscle, resulting in muscle activity. The neuromuscular junction has a specific morphology related to the type of muscle fibre. Structural and functional differences of NMJs are associated with muscle performance [11]. 

Pigeons (*Columba livia domestica*) have been used in sports competitions for a long time and have been exploited during war [12]. New Kim, a Belgian ace, is currently the most expensive carrier pigeon known, with an estimated value of $1.9 million. [13]. Improving the racing performance of pigeons using methods based on phenotypic observation of parental individuals, pedigree analysis, and compiling competition outcomes resulted in the improvement of many important features in this aspect [14]. A pigeon as a good athlete should be distinguished by physical endurance and speed so that it can cover long distances in the shortest possible time. Reliable orientation in space is very important here. The ability of pigeons to return to their loft thousands of kilometers away is one of the most fascinating and enigmatic avian abilities. To this day, it is not entirely clear what the birds are guided by when they return to their home. Nevertheless, when training pigeons, breeders rely on the natural attachment of these birds to their home. 

There are several theories that could explain this ability, including the use of the earth’s magnetic field due to a developed magnetic sense. It has been suggested that cryptochromes located in the retina that function as magnetosensors participate in this, and the process is mediated by the optic nerve [15]. According to another theory, magnetoreception may be based on magnetite-based receptors located in the upper part of the pigeon’s beak, and the trigeminal nerve participates in signal transmission [16,17]. Moreover, the involvement of iron-rich organelles in the sensory cells of avian ear has also been proposed [18]. The last two theories have been later questioned [19,20]. There was also a hypothesis about the existence of electroreceptors participating in the magnetoreception of pigeons [21]. According to a different hypothesis proposed by Papi et al. [22], pigeons are able to return home from unknown places based on the “olfactory map”, i.e., using the mechanism of chemoreception. The role of olfactory cues in navigation were proven using GPS data and an examination of activation of the olfactory system in pigeons. [15,23]. Jorge et al. [24] suggest that some olfactory cues may be used in navigation by older, experienced birds. 

Anjum et al. [25] found CASK expression in retinal synapses. Furthermore, CASK is important for the development of the optic nerve [26,27] and in retinal function [28]. Saavedra et al. [29] found that CASK protein is very important (present in the olfactory cilia) in odour-mediated signal transduction by olfactory sensory neurons. Therefore, this protein may be an important component of the navigation mechanism in pigeons.

Selection of pigeons is focused on the speed of return to the loft in competition flights. In pigeon research, a candidate gene may be proposed, and, using homing and non-homing pigeons (*Columba livia domestica*) and racing records, potential associations between racing performance and DNA polymorphisms can be uncovered. In the group of potential genetic markers of pigeons’ racing performance are the *LDHA* gene [30,31,32,33], *DRD4* [34,35], *FCBP* (*F-KER*) [35,36,37], *CRY1* [38] and *MSTN* [39]. In whole genome analyses of 95 pigeons of various breeds, polymorphisms in the *GSR* and *LRP8* genes have been identified as a potential markers of racing performance in homing pigeons [40,41]. 

In the first genome-wide study in pigeons, Gazda et al. [42] identified several significant signatures of positive selection in the genome of homing pigeons. The strongest signal was located in the *CASK* gene; however, its specific location was not identified. The signature of selection in the *CASK* was stronger close to the promoter region of the gene.

The aim of the study was to detect the nucleotide sequence variability in the calcium/calmodulin-dependent serine protein kinase (*CASK*) gene (including a part of the promoter and first exon) in domestic pigeons and to analyse the potential impact of DNA polymorphisms on the flight performance of homing pigeons.

## 2. Materials and Methods

### 2.1. Animals

The research included a total of 517 pigeons (422 homing pigeons—*Columba livia domestica*, 93 individuals belonging to non-homing breeds—*Columba livia domestica*, and 2 rock pigeons—*Columba livia*). The experiment was divided into two stages—DNA polymorphism detection and association analysis. 

In the first step of the study, 206 animals were included (111 homing pigeons from Belgium, i.e., Natural Antwerp and Aces—two separate groups; 93 non-homing pigeons from local breeder—combined group of Flying and Fancy and Utility; 2 rock pigeons from Prof. Eberhard Haase, Kiel). The details concerning pigeons used in the first stage of the study are presented in Table 1.

The association study (Stage 2) included a total of 311 young homing pigeons (144 females and 167 males), kept in one loft (Kuyavian-Pomeranian voivodeship). Pigeons were fed with the mixture of grains, supplemented with minerals (grits) and vitamins. All individuals (from association study) participated in racing competitions (0344 Rypin, the member of the Polish Association of Racing Pigeon Breeders). 

### 2.2. DNA Analyses

The blood samples (~200 μL) were collected (in 2013–2016) from the metatarsal vein into tubes containing K_3_EDTA (Greiner Bio-One). DNA was isolated using Master Pure^TM^ DNA Purification Kit for Blood (V.2, Epicentre Biotechnologies, Madison, WI, USA). 

At the first stage of the study (i.e., SNP detection), the PCR primers were designed to produce a 361-base pairs (bp) fragment of the *CASK* gene (a part of the promoter and first exon of transcript variants X1–X7, Gene ID: 102088681) using Primer3 software (v. 4.1.0, https://primer3.ut.ee/, accessed on 15 May 2023) [43] and NW_004973256.1 sequence: 

CASKe1-F: 5′-CCAGAAAAGGCTTTGAGGTG-3′

CASKe1-R: 5′-GCCTGGCTCTGTTCTCTTTG-3′.

The primers flanked a DNA fragment from nucleotides 8689 to 9049 of the NW_004973256.1 sequence. 

PCR reaction (in a total volume of 15 µL) contained: ~100 ng of template DNA, 15 pmol of forward and reverse primers, 1 × Dream Taq Buffer (with 2.0 mM MgCl_2_), 0.2 mM dNTP, and 0.3 units of Dream Taq-polymerase. The following temperature profile of the PCR reaction was used: denaturation at 94 °C/5 min, followed by 30 cycles at 94 °C/30 s, annealing of primers at 60 °C/30 s, synthesis of DNA at 72 °C/40 s, and final extension at 72 °C for 5 min.

PCR amplicons from unrelated individuals (two utility pigeons—King and Strasser breeds, three homing pigeons—short, middle, and long-distance pigeons, and one rock pigeon) were sequenced by a specialized third-party company (Genomed, Warsaw, Poland). Chromas software [44] was used to read and analyze the DNA sequencing results. 

One of the detected polymorphic sites (g.8893G > A) was selected for genotyping in all individuals (the SNP is located a very close to the start codon—g.8896). The amplified DNA (361 bp with the same pair of primers) was digested with 3 units of *Alw*26I restriction enzyme for 3 h at 37 °C. The digestion products were separated by horizontal electrophoresis in a 2.5% agarose gels (EUR_X_, 1×TBE) and stained with ethidium bromide. 

### 2.3. Statistical Analysis

The gene and genotype frequencies of the *CASK* gene (g.8893G > A, *CASK*/*Alw*26I) were determined in the groups of domestic pigeons (Natural Antwerp, Aces, and Non-Homing groups from Stage 1, in total, 204 individuals—2 rock pigeons were not included in the statistical analysis). The Hardy–Weinberg equilibrium was tested using the chi-square test, employing the Gene-Calc online tool [45]. Additionally, the pigeon groups (Antwerp, Aces, and Non-Homing) were examined for differences in genotype distributions. In this case, the chi-square test of independence was employed [46]. Differences in genotype proportions between the pigeon groups were assessed using the chi-square test for proportions with Holm correction [47], which adjusted the P-values and was available in the R program [48].

Ace points (AP) were used for measuring the racing performance of pigeons tested [17]. The data set consisted of 1111 race records (four races). 

The effect of the SNP g.8893G > A (*CASK*/*Alw*26I) on the value of ace points was estimated using the following ANOVA model:

y_ijklm_ = μ + G_i_ + S_j_ + R_k_ +p_l_ + GS_ij_ + GR_ik_ + e_ijklm_,

where y_ijklm_—the mean value of ace points, μ—the overall mean for the trait, G_i_—the effect of the genotype, S_j_—the effect of the sex, R_k_—the effect of the race, p_l_—the random effect of the individual, GS_ij_—the interaction genotype x sex, GR_ik_—interaction genotype x race, and e_ijklm_—the random error.

The influence of individual factors within each flight was also analysed separately according to the ANOVA model:

y_ijk_ = μ + G_i_ + S_j_ + GS_ij_ + e_ijk_,

where y_ijk_—the mean value of ace points, μ—the overall mean for the trait, G_i_—the effect of the genotype, S_j_—the effect of the sex, GS_ij_—the interaction genotype x sex, and e_ijk_—the random error.

The assumptions of ANOVA applicability were fulfilled; normal distribution in each level of the analysed effects was present according to the Shapiro–Wilk test (*p* > 0.01), homogeneity of variance in each level of the examined factors was present according to Levene’s test (*p* > 0.01).

Tukey’s post hoc test was used in the ANOVA method to check the significance of differences between the levels of the analysed factors.

## 3. Results

**Figure 1:**
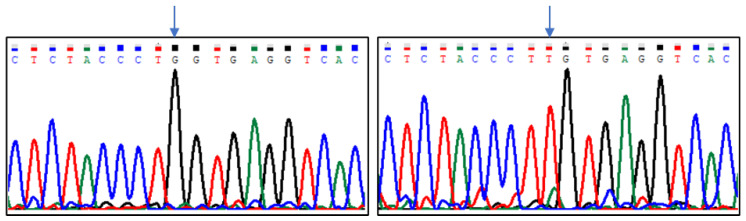
The results of sequencing analysis: SNP1 (g.8838G > T); (**left**)—*CASK^GG^* genotype, (**right**)—*CASK^TT^* genotype (an arrow shows the polymorphic site).

**Figure 2:**
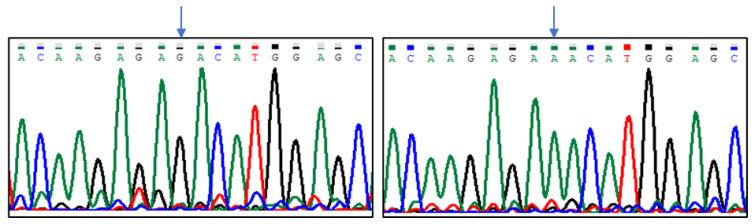
The results of sequencing analysis: SNP2 (g.8893G > A); (**left**)—*CASK^GG^* genotype, (**right**)—*CASK^AA^* genotype (an arrow shows the polymorphic site).

**Figure 3:**
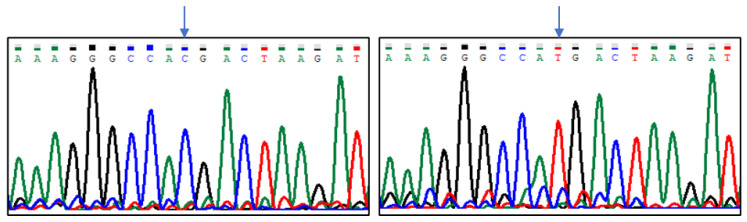
The results of sequencing analysis: SNP3 (g.8915C > T); (**left**)—*CASK^CC^* genotype, (**right**)—*CASK^TT^* genotype (an arrow shows the polymorphic site).

**Figure 4:**
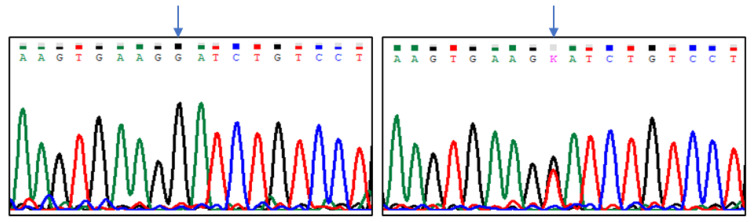
The results of sequencing analysis: SNP4 (g.8941G > T); (**left**)—*CASK^GG^* genotype, (**right**)—*CASK^GT^* genotype; K = G or T (an arrow shows the polymorphic site).

**Figure 5:**
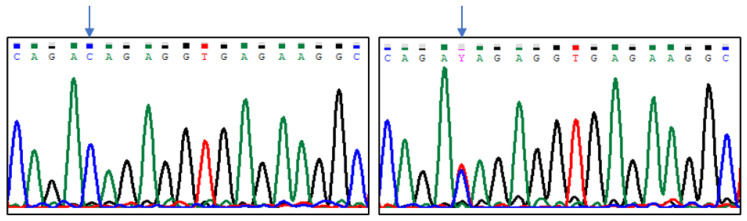
The results of sequencing analysis: SNP5 (g.8986C > T); (**left**)—*CASK^CC^* genotype, (**right**)—*CASK^GT^* genotype; Y = C or T (an arrow shows the polymorphic site).

Sequencing results indicated five polymorphic sites in the analysed region of the *CASK* gene, which were located at positions (Gene ID: 102088681, NW_004973256.1): g.8838G > T, g.8893G > A, g.8915C > T, g.8941G > T, and g.8986C > T (Figure 1, Figure 2, Figure 3, Figure 4 and Figure 5). The first two SNPs are located at positions −59 and −3, relative to the transcription start site. The three last SNPs are located at positions +17, +43, and +88 relative to the transcription start site. As g.8915C > T is the T17M amino acid change at the polypeptide level, g.8941G > T generates the D43Y amino acid change, while g.8986C > T introduces premature codon termination (Q88X).

**Figure 6:**
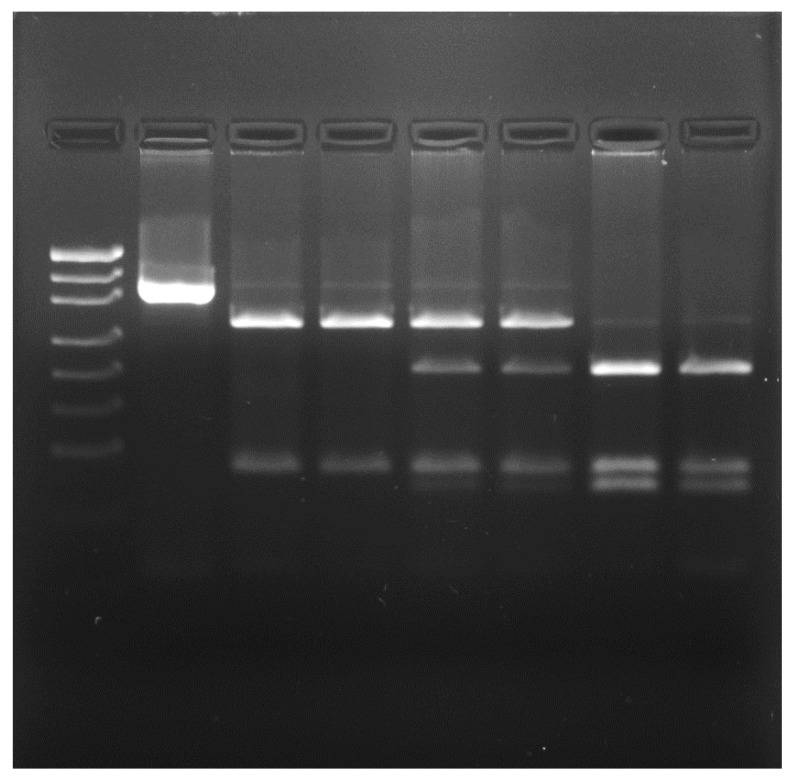
Determination of *CASK/Alw*26I genotypes; from left to right: lane 1—DNA ladder (pUC19/*Msp*I), lane 2—PCR product, lanes 3 and 4—*CASK/Alw*26I*^AA^*, lanes 5 and 6—*CASK/Alw*26I*^AG^*, lanes 7 and 8—*CASK/Alw*26I*^GG^*.

In the course of PCR-RFLP (SNP g.8893G > A) genotyping, the following DNA restriction fragments were observed: 279 and 82 bp for the *CASK/Alw*26I*^AA^* genotype, 279; 197, 82, and 82 bp for the *CASK/Alw*26I*^AG^;* and 197, 82, and 82 bp for the *CASK/Alw*26I*^GG^* (Figure 6).

The frequencies of *CASK/Alw*26I (SNP g.8893G > A) genotypes and alleles are presented in Table 1 and Table 2. The results of genotyping in the first group of pigeons (Stage 1, Table 2) showed that there were significant associations between genotype and pigeon groups (χ^2^ = 58.82, df = 4, *p* < 0.05). Within the *AA* genotype, the proportions differed among the pigeon groups (χ^2^ = 27.15, df = 2, *p* < 0.05). Between the homing (Natural Antwerp) group and the non-homing group, a chi-square value of 17.028 was observed (df = 1, *p* < 0.05). A difference in proportions was also found between the homing (Aces) group and the non-homing group (χ^2^ = 22.294, df = 1, *p* < 0.05). 

Distribution of *CASK* genotypes (g.8893G > A) in the group of homing pigeons used in association study (Stage 2) was consistent with Hardy–Weinberg’s law (Table 3). 

The association between the SNP (g.8893G > A) and racing performance of young pigeons was further examined. The average values of AP for *CASK* genotypes and pigeon sex are given in the Table 4. 

The effect of the next race was statistically significant (*p* < 0.05, Table 5), as was the random effect of the pigeon (*p* < 0.05). The difference in average AP values in the next race was statistically significant, except for races 1 and 2 (Table 5).

The average values of ace points in studied races, stratified by *CASK* genotype and pigeon sex are presented in Table 6 and Table 7. The interactions of GS (genotype-sex) and GR (genotype-race) were not statistically significant. 

Only in flight number 4 (Table 7) was the influence of pigeon sex statistically significant (*p* < 0.05).

## 4. Discussion

A properly functioning *CASK* gene may be important in the development and maintenance of body functions that are crucial from the point of view of homing pigeon breeding. The studied gene appears to be highly polymorphic as indicated earlier by Stefaniuk-Szmukier et al. [49]. The present study confirmed the existence of five SNPs in part of the promoter and first exon of the *CASK* gene in analysed pigeons. Three of them turned out to be transitions, which is consistent with the observation of their greater frequency in the genome, as compared to transversions [50]. The substitutions T17M and D43Y resulting from the presence of g.8915C > T and g.8941G > T changes, respectively, as well as the nonsense mutation g.8986C > T, are located within the CASK CaMK domain, which is important for complexes with Mint1 and Liprin-α2, two prominent presynaptic partners of CASK. Thus, they could change the ability of CASK interaction with these proteins. Moreover, the C-terminus of neurexin is the known in vivo substrate for the CASK CaM kinase activity, suggesting the possibility that alterations in the CaMK domain might affect the interaction between both proteins [51,52]. It is therefore assumed that these amino acid changes might affect synaptic function, including odour-mediated signal transduction or electrical properties of neurons. However, confirmation of the functionality of the above SNPs in terms of pigeon flight results requires separate research.

The region flanking the start site of the sequences being translated plays a key role in the initiation of this process, and is of particular importance in recognition of the start site by the ribosome scanning complex [53]. The presence of a purine in the −3 position and a guanine in the +4 position are important for efficient translation initiation [54]. It has been shown that SNPs occurred at and around the translation start site, and this can have a significant impact on start site recognition and, thus, the assembly of the translation machinery [55,56]. The g.8893G > A polymorphism, located 3 bp upstream from the transcription start site, seems to be related to such functionality. Therefore, it was the subject of a more detailed analysis in our study. A significant difference in the frequency of the *AA* genotype was found between homing and non-homing pigeons groups, with a large predominance in homing pigeons. Moreover, this genotype has not been found in rock pigeons. However, the obtained results were from two individuals only; therefore, they are not reliable and analysis should be carried out on a larger group of rock pigeons. Nevertheless, there is an assumption about the role of the examined SNP in shaping the variability of traits related to the homing ability of pigeons. A significant increase in the frequency of the *A* allele in the homing pigeon may be the result of many years of selection for the improvement of racing performance.

Homing pigeons are interesting subjects in which the genetic basis of traits affecting racing performance can be sought. Research is being conducted to detect polymorphic sites in genes important from this point of view and to search for associations between SNPs and racing performance of pigeons. Recently, a comparative analysis of the whole genomes of homing and non-homing pigeons contributed to the selection of candidate genes for shaping racing phenotypes. Among them are those that are involved in the development and functioning of the central nervous system [40,42]. An example is the *CASK* gene, which is involved in the development of neuromuscular junctions. Many signatures of positive selection within the genome of the homing pigeon were obtained, and the strongest one coincided with, among others, the promoter region of the *CASK* gene. However, no diagnostic alleles were detected between homing and non-homing pigeons. The authors suggest that the traits that enable fast flight, endurance, and precise navigation in pigeons are possibly of a polygenic nature [42].

The navigational abilities of the pigeons seems to be important during competition. However, the participation of a pigeon in the competition is undoubtedly a large physical effort. Such activity is known to cause changes, both at physiological and biochemical as well as molecular levels. Changes depend on the duration and intensity of physical exercise. The start of contractile activity results in a rapid increase in the level of Ca^2+^ in the cells [57,58]. Muscle contraction and subsequent metabolic changes contribute to the activation of several kinases and phosphatases crucial in signal transduction. Signalling pathways dependent on changes in Ca^2+^ concentration are of particular importance. They include e.g., Ca^2+^/calmodulin-dependent kinase II and Ca^2+^-dependent protein kinase C [57,59]. It should be noted that the calcium-binding troponin C plays a dominant role in the mechanism of striated muscle contraction, while calmodulin (CaM) is involved in the regulatory functions of this process [58].

Calmodulin, as a calcium level sensor, plays an important role in the activation of calcium-dependent signalling pathways, important in maintaining normal cellular functions [60]. It has been found that some mutations in the gene encoding CaM in humans can result in arrhythmia [61]. CaM, as a molecule without enzymatic activity, can interact with various enzymes, affecting their activation [62,63]. Its targets include calmodulin-dependent kinases [64]. One of them is CASK—calcium/calmodulin (CaM)-dependent serine protein kinase. It is a member of the membrane-associated guanylate kinase (MAGUK) family of scaffold proteins and is characterized by a multi-domain structure [65]. As a member of this family, CASK participates in the assembly of multi-protein complexes involved in tissue development and cell signalling [66]. In addition to the family-specific motifs [67], CASK contains a Ca^2+^/CaM-dependent protein kinase (CaMK) domain with high sequence identity to the Ca^2+^/CaM-dependent protein kinase II (CaMKII) domain [4,68]. Consequently, it has the ability to bind various proteins [69]. An example is the formation of a complex with two scaffold proteins (Veli and Mint1) in the mammalian central nervous system [5], which may be involved in the modulation of synaptic transmission [68]. Its ability to interact with neurexin has been also demonstrated [4]. Due to the lack of critical residues for Mg^2+^-ATP binding, CASK can function as an Mg^2+^-independent kinase with very low kinase activity, capable of phosphorylating neurexin [51]. 

CASK is a molecule highly expressed in neurons in various regions of the brain and elsewhere in the nervous system [70]. In addition to participating in the regulation of synaptic functions [62], it has been found to be involved in regulating the expression of genes associated with the development of neurons [70]. The CASK-neurexin interaction has also been shown to be crucial for the development of the optic nerve [26,27]. CASK protein is expressed in retinal synapses [25], and interactions mediated by its CaMK domain may play an important role in retinal function [28]. Moreover, CASK protein is found in the olfactory cilia fraction, which suggests a role in the organization of an odour transduction complex [29]. The location of CASK in pre- and postsynaptic NMJ membranes has also been described, and the interaction of CASK with neurexin helps to stabilize this junction [8]. Furthermore, studies by Gardner et al. [9] suggest a dual role of CASK in the NMJ components, such as skeletal muscle and motor neuron development.

Mutations in the *CASK* gene in fruit flies (*Drosophila melanogaster*) resulted in behavioural disorders and abnormal neurotransmitter release [71]. Moreover, the odour-induced Ca^2+^ signaling in mushroom bodies (learning centers of *Drosophila*) was reduced in CASK knockdown larvae [72]. In mice, a gene deletion led to perinatal death [7]. Mutations in the *CASK* gene in humans have been associated with X-linked intellectual disability (XL-ID), as well as pontine and cerebellar hypoplasia (MICPCH), with considerable phenotypic variability [73]. People with MICPCH are additionally characterized by eye abnormalities, such as optic nerve hypoplasia and retinopathy [28]. Moreover, they have hearing loss caused by nerve problems in the inner ear. In addition, such people may have muscle hypotonia in the torso, as well as hypertonia and (spasticity in the limbs, which can result in mobility problems [74].

In the presented study, no significant associations were found between the genotype and the mean values of AP points obtained by the pigeons in the competitions. Nevertheless, it seems that homing pigeons with the *AA* genotype might have potential to score more points than the pigeons with the *GG* genotype. However, this requires confirmation in further research. 

## 5. Conclusions

In this study, out of five detected polymorphic sites within the *CASK* gene, one was included in the analysis of the potential impact on the racing performance of pigeons. The obtained research results seem promising, despite the lack of statistical confirmation. They can be treated as a contribution to further research that could confirm the functionality of the analysed g.8893G > A SNP in shaping the sports phenotype of homing pigeons. The *AA* genotype could be useful as a selection criterion in pigeon breeding.

## Figures and Tables

**Table 1:** Characteristics of the studied pigeons (Stage 1 of the study).

Group of Pigeons			
Homing		Non-Homing	
Natural Antwerp (*n* = 82)	Aces (PiGen vof) (*n* = 29)	Flying and Fancy (*n* = 58)	Utility (*n* = 35)
Janssen (40) Bricoux (2) De Smet-Matthys (9) Grondelaers (5) Meulemans (6) Stichelbaut (10) Wanroy (10)		G. Show Homer (6) Bagdad of Nuremberg (4) G. Magpie (5) G. Long Faced Tumbler (10) Polish Barb (4) Vienna Kiebitz (1) Danzig Highflier (9) Carrier (6) Polish Krymka Tumbler (3) Polish Owl (1) Fantail (4) Polish Short-Beaked (5)	King (11) Mondain (4) Strasser (10) Maltese (3) Polish Lynx (3) Cauchois (4)

G.—German; due to the small number (2 individuals), rock pigeons were not included in the statistical analysis.

**Table 2:** Frequencies of genotypes and alleles for SNP2 (g.8893G > A, *CASK*/*Alw*26I) in individual pigeon groups with significant differences in proportions between pigeon groups within genotypes (chi-square test for proportions).

Group	*n*	Genotypes			Alleles	
		*AA*	*AG*	*GG*	*A*	*G*
Homing pigeons (Natural Antwerp)	82	0.488 ^a^ (*n* = 40)	0.463 (*n* = 38)	0.049 (*n* = 4)	0.720	0.280
Homing pigeons (Aces, PiGen vof)	29	0.655 ^a^ (*n* = 19)	0.345 (*n* = 10)	- (*n* = 0)	0.792	0.208
Non-homing pigeons	93	0.194 ^b^ (*n* = 18)	0.344 (*n* = 32)	0.462 (*n* = 43)	0.366	0.634
Total	204	0.384 (*n* = 77)	0.388 (*n* = 80)	0.228 (*n* = 47)	0.578	0.422

Chi-square test for proportions with Holm’s correction; different letters indicate statistically significant differences in proportions at *p* < 0.05.

**Table 3:** Frequencies of genotypes and alleles for SNP2 (g.8893G > A, *CASK*/*Alw*26I) in the homing pigeon group with the Hardy–Weinberg equilibrium (HWE).

Group	*n*	Genotypes			Alleles		HWE (*p*-Value)
		*AA*	*AG*	*GG*	*A*	*G*	
Homing pigeons (A.S.)	311	0.502 (*n* = 156)	0.434 (*n* = 135)	0.064 (*n* = 20)	0.719	0.281	*p* > 0.05

A.S.—association study; *p* > 0.05 suggested the population conforms to Hardy–Weinberg equilibrium.

**Table 4:** Differences in ace points (AP) between *CASK* genotypes and pigeon sexes (ANOVA).

Genotype/Sex	RR	AP	SE	ANOVA Test
*AA*	552	29.16	1.62	*p* > 0.05
*AG*	482	27.42	1.68	
*GG*	77	24.02	4.20	
Females	511	28.59	1.67	*p* > 0.05
Males	600	27.58	1.52	

RR—number of race records; SE—standard error of the mean.

**Table 5:** Differences in ace points (AP) between pigeon flights (ANOVA).

Race Number	RR	AP	SE	ANOVA Test
1	227	27.14 ^a^	2.32	*p* < 0.05
2	298	21.82 ^a^	2.03	
3	289	49.44 ^b^	2.05	
4	297	14.16 ^c^	2.03	

RR—number of race records; SE—standard error of the mean; values in column marked with different superscript letter indicate statistically significant differences at *p* < 0.05.

**Table 6:** Differences in ace points (AP) between the groups genotype × flight (ANOVA).

Number of Race	Genotype	RR	AP	SE
1	*AA*	110	28.76	3.33
	*AG*	99	27.89	3.55
	*GG*	18	16.81	8.28
2	*AA*	150	23.73	2.81
	*AG*	128	20.13	3.05
	*GG*	20	19.27	7.73
3	*AA*	145	49.46	3.51
	*AG*	125	50.73	3.77
	*GG*	19	41.82	9.67
4	*AA*	147	14.87	2.27
	*AG*	130	12.86	2.41
	*GG*	20	15.40	6.17

RR—number of race records; SE—standard error of the mean.

**Table 7:** Differences in ace points (AP) between the groups flight × sex (ANOVA).

Number of Race	Sex	RR	AP	SE
1	Female	102	23.82	4.77
	Male	125	25.15	4.27
2	Female	140	19.73	4.32
	Male	158	22.36	3.94
3	Female	133	48.31	5.31
	Male	156	46.37	5.01
4	Female	136	8.88 *	3.46
	Male	161	19.87 *	3.14

RR—number of race records; SE—standard error of the mean; *—statistically significant differences at *p* < 0.05.

## Data Availability

Data is contained within the article.
